# Datenverarbeitung in sicheren Verarbeitungsumgebungen am Beispiel von MRT-Gehirnscans – zugleich ein Plädoyer für eine rechtssichere Forschungsgrundlage

**DOI:** 10.1007/s00103-023-03807-z

**Published:** 2023-12-14

**Authors:** Louisa Specht-Riemenschneider, Bernadette Heineking

**Affiliations:** Lehrstuhl für Bürgerliches Recht, Recht der Datenwirtschaft, des Datenschutzes, der Digitalisierung und der Künstlichen Intelligenz, Rheinische Friedrich-Wilhelms-Universität Bonn, Adenauer-Allee 46a, 53113 Bonn, Deutschland

**Keywords:** Datentreuhänder, In-situ Datenverarbeitung, Datenverarbeitung zu Forschungszwecken, Weiterverarbeitung, Radiologiedaten, Data trustee, In situ data processing, Data processing for scientific research purposes, Further processing, Radiological data

## Abstract

Die Nutzung von Datenbeständen für medizinische Forschung bietet großes gesamtgesellschaftliches Potenzial, können sich doch durch die Auswertung großer Datenmengen mit maschinellen Lernverfahren teils ganz neue Forschungsansätze, Diagnose- und Behandlungsmethoden ergeben. Häufig scheitert eine solche Nutzung von Datenbeständen aber an den hohen Voraussetzungen oder unklaren Anforderungen des Datenschutzrechts.

Gerade die Verarbeitung visueller Gesundheitsdaten wie MRT-Gehirnscans geht mit besonderen Risiken für die betroffenen Personen einher, die die rechtmäßige Verarbeitung dieser Daten zu Forschungszwecken erschweren. Zur Reduzierung dieser Risiken können als unabhängige Anonymisierungs- und Pseudonymisierungsinstanz eingesetzte Datentreuhänder beitragen, die eine sichere Verarbeitungsumgebung bereitstellen, in der Gesundheitsdaten kurzfristig gespeichert, ausgewertet und anschließend gelöscht werden.

Diese Risikoreduktion trägt zur Rechtmäßigkeit der Datenverarbeitung bei, führt sie doch dazu, dass Abwägungsentscheidungen, wie z. B. nach Art. 9 Abs. 2 lit. j, Art. 89 Abs. 1 Datenschutzgrundverordnung (DSGVO), § 6 Abs. 2 Gesundheitsdatenschutzgesetz Nordrhein-Westfalen (GDSG NW), § 17 Datenschutzgesetz Nordrhein-Westfalen (DSG NRW) bzw. § 27 Bundesdatenschutzgesetz (BDSG), oder die Beurteilung der Kompatibilität des Sekundärzwecks der Verarbeitung mit dem Erhebungszweck eher zugunsten der Datenverarbeitung ausfällt. Der Einsatz von Datentreuhandmodellen kann insofern auch zum Abbau datenschutzrechtlicher Hürden der Verarbeitung solcher Gesundheitsdaten zu wissenschaftlichen Forschungszwecken beitragen.

## Abstract (en)

The use of data for medical scientific research offers great potential for society as a whole, as the evaluation of large volumes of data with machine learning methods can result in new research approaches as well as new methods of diagnostics or treatment. However, the use of such data often fails due to high prerequisites or unclear requirements of data protection law.

Processing of radiology data, such as MRI brain scans, is tied to specific risks for data subjects. This complicates the processing of such data for research purposes. Data trustees can help to reduce these risks through offering independent anonymization and pseudonymization services as well as secure processing environments in which health data is stored only for the time required for processing and analysis and is subsequently deleted.

Thus, the use of data trustees can help to comply with data protection law, with risk-reduction being considered in favor of processing in decisions weighing the interests for and against data processing as necessary to comply with Art. 9 Para. 2 lit. j, Art. 89 Para 1 of the General Data Protection Regulation (GPDR) in conjunction with national law, or when assessing the compatibility of the secondary purpose of the processing with the purpose of collection. In this respect, the use of data trust models can contribute to the reduction of hurdles for the data processing of health data for scientific research purposes.

## Einleitung

Die Analyse großer Datenbestände hat das Potenzial, die medizinische Forschung ganz erheblich voranzubringen und Therapiemöglichkeiten auf neue Grundlagen zu stellen. Dies gilt nicht nur für die Auswertung von Laborbeständen oder Routinedaten von Patientinnen und Patienten, sondern auch und gerade für Bilddaten aus der Radiologie oder der Pathologie. Beispielsweise können über Magnetresonanztomographie (MRT) erstellte Aufnahmen von Gehirnquerschnitten für die Grundlagenforschung sowie die epidemiologische Erforschung von Krankheitsbildern bis hin zur Entwicklung von Mechanismen künstlicher Intelligenz (KI) zur verbesserten Diagnostik herangezogen werden. Gerade Letzteres erfordert große Datenmengen, mit denen die KI-Software trainiert werden kann.

Derzeit scheitert die Bereitstellung und Nutzung von Patientendatenbeständen zu (kooperativen) wissenschaftlichen Forschungszwecken vielfach an den hohen und teils unklaren datenschutzrechtlichen Anforderungen. Gesundheitsdaten im Sinne des Art. 4 Nr. 15 Datenschutzgrundverordnung (DSGVO) unterliegen strengen Schutzanforderungen nach Art. 9 DSGVO. Diese werden auf Grundlage der Öffnungsklauseln des Art. 9 Abs. 2 lit. a–j, Abs. 4 DSGVO durch Vorgaben des Bundesdatenschutzgesetzes (BDSG), der Landesdatenschutzgesetze (LDSG) sowie der Landeskrankenhausgesetze (LKHG) oder Landesgesundheitsdatenschutzgesetze (LGDSG) ergänzt.^1^

In den LKHG und LGDSG werden besondere Vorgaben für die Verarbeitung von Patientendaten normiert. Dieses bereichsspezifische Recht gilt grundsätzlich vorrangig gegenüber dem BDSG [3], das im Allgemeinen für die Datenverarbeitung durch öffentliche Stellen des Bundes sowie Private, also natürliche oder juristische Personen des Privatrechts, Anwendung findet, und gegenüber den LDSG, die für die Datenverarbeitung durch öffentliche Stellen der Länder Anwendung finden. Damit gelten in den Bundesländern jeweils einheitliche Vorgaben der LKHG bzw. LGDSG für die Datenverarbeitung in Krankenhäusern, unabhängig von ihrer Trägerschaft.^2^

Die Einwilligung der Patientinnen und Patienten, die nach Art. 9 Abs. 2 lit. a DSGVO die Datenverarbeitung rechtfertigen kann, ist in der Regel nicht oder nur äußerst schwer zu generieren, muss sie doch für einen konkreten Zweck eingeholt werden, im Rahmen von wissenschaftlichen Forschungsvorhaben ist eine hinreichend konkrete vorherige Zweckbestimmung aber vielfach nicht möglich. Auch das Einwilligungsformular der Medizininformatik-Initiative (MII) mit seiner Broad-Consent-Lösung, die sogenannte erweiterte Einwilligung, hilft aufgrund ihrer Komplexität vielfach nicht.^3^

Aber auch auf gesetzlicher Grundlage ohne Einwilligung ist die Datenverarbeitung zu Forschungszwecken nach Art. 9 Abs. 2 lit. j DSGVO in Verbindung mit nationalem Recht, § 27 BDSG sowie Parallelvorschriften im Landesrecht wie § 17 DSG NRW und § 6 GDSG NW gestattet. Allerdings ist hier eine Abwägung zwischen Betroffenen- und Verarbeitungsinteressen für jedes einzelne Datum erforderlich, die angesichts der Sensibilität von Patientendaten und der (zusätzlichen) Risiken, die gerade mit der Datenverarbeitung durch Dritte einhergehen, vielfach zugunsten der oder des Betroffenen ausfällt oder zumindest nicht rechtssicher abgesehen werden kann.

Ebendies gilt auch für die Prüfung der Vereinbarkeit von Primär- und Sekundärzwecken, die erforderlich ist, wenn Daten zu anderen Zwecken verarbeitet werden sollen als denjenigen, zu denen sie erhoben wurden. Wenngleich die Förderung der wissenschaftlichen Forschung ein unionsrechtlich gefördertes Ziel ist, Art. 179 des Vertrags über die Arbeitsweise der Europäischen Union (AEUV), das auch im Kontext der DSGVO Anklang findet, vgl. Erwägungsgrund (ErwGr.) 159 S. 3 DSGVO, unterbleibt deshalb häufig auch die gesellschaftlich wünschenswerte Datenverarbeitung im Rahmen von medizinischen Forschungsvorhaben, die zu unser aller Nutzen erfolgen würde.

Abhilfe schaffen könnten Datentreuhänder, die zur Reduzierung der Verarbeitungsrisiken durch technisch-organisatorische Maßnahmen beitragen. Beispielsweise könnten solche Datentreuhänder sichere Verarbeitungsumgebungen im Sinne abgetrennter, durch technische und organisatorische Maßnahmen vor unbefugten Verarbeitungstätigkeiten oder unerlaubtem Zugriff gesicherter virtueller „Räume“ bereitstellen,^4^ in denen Gesundheitsdaten unter kurzfristiger Speicherung verarbeitet und anschließend gelöscht werden. Diese Maßnahmen können sich in der erforderlichen Abwägung ausschlaggebend für ein Überwiegen der Verarbeitungsinteressen auswirken.

Die rechtlichen Anforderungen an die einwilligungsfreie Erhebung und Verarbeitung von Patientendaten werden im Rahmen dieses Aufsatzes am Beispiel von MRT-Gehirnscans erörtert, einschließlich eines Ausblicks auf mögliche Vorteile, die der Einsatz solcher Datentreuhänder vor diesem Hintergrund mit sich bringen könnte.

Im Folgenden wird zunächst ein Überblick über die rechtlichen Vorgaben für die Verarbeitung von MRT-Bilddaten gegeben, bevor die Rechtmäßigkeit der Datenverarbeitung bei der Erhebung für Behandlungszwecke und für medizinische Forschungszwecke sowie der Weiterverarbeitung für konkrete Forschungsvorhaben dargestellt wird. Anschließend werden im Ausblick die Rechtmäßigkeitsanforderungen unter Einbindung eines Datentreuhänders betrachtet, bevor der Beitrag mit einer Zusammenfassung der Ergebnisse schließt.

## Datenschutzrechtliche Anforderungen an die Verarbeitung von MRT-Bilddaten

Die Verarbeitung personenbezogener Daten richtet sich maßgeblich nach der DSGVO, die auf europäischer Ebene einen einheitlichen Standard für den Schutz und die Verarbeitung personenbezogener Daten bestimmt. Personenbezogene Daten sind nach der Legaldefinition des Art. 4 Nr. 1 DSGVO Informationen, die sich auf identifizierte oder identifizierbare natürliche Personen beziehen. MRT-Bilder sind auch ohne eine Hinzuziehung weiterer (personenbezogener) Metadaten wie Patientenname, Geburtsdatum, Adresse oder Geschlecht personenbezogene Daten im Sinne des Art. 4 Nr. 2 DSGVO. Denn aus den Querschnitten der Gesichtspartie lassen sich 3D-Modelle vollständiger Gesichter (re)konstruieren, die unter Einsatz von Gesichtserkennungssoftware sowie einem Abgleich mit (öffentlich verfügbaren) Bildquellen gezielt einzelnen Personen zugeordnet werden können [7].

### Gesundheitsdaten.

Weil solche MRT-Gehirnscans jedenfalls dem geschulten Betrachter Rückschlüsse über den früheren, gegenwärtigen oder auch zukünftigen Gesundheitszustand der abgebildeten Person ermöglichen, sind sie Gesundheitsdaten im Sinne des Art. 4 Nr. 15 DSGVO.

### Biometrische Daten.

MRT-Gehirnscans sind aber keine biometrischen Daten im Sinne des Art. 4 Nr. 14 DSGVO. Zwar wird zu deren Erstellung ein automatisiertes standardisiertes Verfahren verwendet. Dessen Zweck ist aber nicht spezifisch die Vermessung und Analyse von Merkmalen der Person, um durch Abgleich mit bereits erfassten Referenzmustern zur eindeutigen Identifizierung verwendet zu werden [8]. Ausgehend vom Wortlaut des Art. 4 Nr. 14 DSGVO („mit speziellen technischen Verfahren gewonnene personenbezogene Daten“) sowie des ErwGr. 51 S. 3 („wenn sie mit speziellen technischen Mitteln verarbeitet werden“) liegen biometrische Daten im Sinne des Art. 4 Nr. 14 DSGVO aber nur vor, wenn und soweit personenbezogene Daten mit entsprechenden spezifischen Verfahren verarbeitet werden [9].

Zwar besteht ein Risiko einer solchen Verarbeitung von MRT-Daten [10]. Dieses Risiko genügt aber nicht, um MRT-Bilder als biometrische Daten einzuordnen, es sei denn, sie werden tatsächlich in einem entsprechenden Verfahren zur biometriebasierten Identifizierung natürlicher Personen verwendet. Dieses Risiko ist jedoch bei der Beurteilung der datenschutzrechtlichen Rechtmäßigkeit der Verarbeitung zu berücksichtigen.

## Rechtmäßigkeit der Datenerhebung

Für die Verarbeitung von Gesundheitsdaten gelten nach Art. 9 Abs. 1 DSGVO besonders strenge Anforderungen; ihre Verarbeitung ist grundsätzlich untersagt, es sei denn, die Voraussetzungen eines Ausnahmetatbestands nach Art. 9 Abs. 2 DSGVO liegen vor. In der Literatur ist umstritten, ob Art. 9 Abs. 2 lit. a–j DSGVO im Verhältnis zu Art. 6 Abs. 1 lit. a–f DSGVO eigenständige, speziellere Erlaubnistatbestände normieren [11, 12] oder aber als zusätzliche Voraussetzungen der allgemeinen Erlaubnistatbestände des Art. 6 Abs. 1 DSGVO aufzufassen sind [13, 14].^5^ Für die hier betrachtete Verarbeitung kommt es hierauf allerdings nicht an, denn wenn die strengen Voraussetzungen des Art. 9 Abs. 2 lit. a–j DSGVO gegeben sind, werden in der Regel auch die allgemeineren Bestimmungen der Art. 6 Abs. 1 lit. a–f DSGVO miterfüllt sein [15].

Im Folgenden soll zunächst dargestellt werden, unter welchen Voraussetzungen MRT-Gehirnscans erhoben werden dürfen, und anschließend, welche Anforderungen an eine Weiterverarbeitung zu stellen sind.

### Datenerhebung für Behandlungszwecke

Eine zentrale Rechtsgrundlage für die Erhebung von Gesundheitsdaten für Behandlungszwecke bildet die Einwilligung der Patientin oder des Patienten als betroffene Person nach Art. 9 Abs. 2 lit. a, (Art. 6 Abs. 1 lit. a), Art. 4 Nr. 11, Art. 7 DSGVO. Qualifizierend gegenüber Art. 6 Abs. 1 lit. a DSGVO fordert Art. 9 Abs. 2 lit. a DSGVO die Ausdrücklichkeit der Einwilligung, woraus sich höhere Anforderungen an die Art und Weise, wie eine Person ihre Zustimmung zum Ausdruck bringt, an die Zweckbestimmung sowie an die Information der oder des Betroffenen ergeben [16].

Art. 9 Abs. 2 lit. a DSGVO eröffnet zudem den Mitgliedsstaaten die Möglichkeit, weitere Anforderungen an die Einwilligung zu normieren.^6^ Besondere Formanforderungen an die Einwilligung in die Verarbeitung von Patientendaten und die dahingehende Information trifft etwa auch § 4 Abs. 1 GDSG NW, wonach die Schriftform der Einwilligung erforderlich ist, soweit nicht wegen besonderer Umstände eine andere Form angemessen ist.

Daneben sind die Erhebung und anschließende Verarbeitung von MRT-Bildern als Gesundheitsdaten zu Behandlungszwecken auch ohne Einwilligung möglich, insb. auf Grundlage des Art. 9 Abs. 2 lit. h und lit. i DSGVO in Verbindung mit mitgliedsstaatlichem Recht, etwa §§ 630a ff., 630 f. Bürgerliches Gesetzbuch (BGB) sowie § 10 Abs. 1 lit. a GDSG NW [2].

### Datenerhebung für wissenschaftliche Forschungszwecke

Sollen MRT-Gehirnscans initial zu wissenschaftlichen Forschungszwecken erhoben werden, kommt als Rechtsgrundlage ebenfalls die Einwilligung nach Art. 9 Abs. 2 lit. a, (Art. 6 Abs. 1 lit. a), Art. 7, Art. 4 Nr. 11 DSGVO in Verbindung mit §§ 10 Abs. 2, 6 Abs. 1, 4 GDSG NW in Betracht. Gegebenenfalls kann hierfür das Formular für den Broad Consent der MII genutzt werden (s. oben, Einleitung). Vorteil dieses Einwilligungsformulars ist es, dass die Einwilligung in die Datenverarbeitung zu wissenschaftlichen Forschungszwecken nicht für einen ganz konkreten Zweck erteilt werden muss, sondern für bestimmte Forschungsbereiche oder Teile von wissenschaftlichen Forschungsvorhaben abgegeben werden kann. Die strengen Bestimmtheitsanforderungen an die Zweckbestimmung der Datenverarbeitung vor der Erhebung erfahren insoweit unter Berücksichtigung des ErwGr. 33 DSGVO eine gewisse Lockerung. Dies erleichtert die Datenverarbeitung zu wissenschaftlichen Forschungsvorhaben, bei denen zum Zeitpunkt der Datenerhebung die konkreten Verarbeitungszwecke (noch) nicht genau bestimmt werden können [18].

Ohne Einwilligung sind die Erhebung und Verarbeitung von Gesundheitsdaten zu wissenschaftlichen Forschungszwecken nach Art. 9 Abs. 2 lit. j DSGVO auf Grundlage von mitgliedsstaatlichem Recht gestattet. Solche Vorschriften haben nach Art. 9 Abs. 2 lit. j DSGVO in angemessenem Verhältnis zu dem verfolgten Ziel zu stehen, den Wesensgehalt des Rechts auf Datenschutz zu wahren und angemessene und spezifische Maßnahmen zur Wahrung der Grundrechte und Interessen der betroffenen Person vorzusehen.

Art. 89 Abs. 1 DSGVO normiert weitere Anforderungen an geeignete Garantien zum Schutz der Rechte und Freiheiten natürlicher Personen bei der Verarbeitung zu wissenschaftlichen Forschungszwecken, insb. zur Achtung des Grundsatzes der Datenminimierung (Art. 5 Abs. 1 lit. c, Art. 89 Abs. 1 S. 2 DSGVO) durch zeitnahe Pseudonymisierung (Art. 89 Abs. 1 S. 3 DSGVO) und Anonymisierung (Art. 89 Abs. 1 S. 4 DSGVO).

Die Anforderungen der Art. 9 Abs. 2 lit. j, Art. 89 Abs. 1 DSGVO wurden mit § 27 Abs. 1 S. 1 BDSG im nationalen Recht umgesetzt, der eine Rechtsgrundlage für die Verarbeitung besonderer Datenkategorien zu wissenschaftlichen Zwecken unter Ergreifung zusätzlicher Maßnahmen nach § 27 Abs. 1 S. 2, § 22 Abs. 2 S. 2 BDSG vorsieht [19]. Parallele Vorgaben auf Landesebene trifft etwa § 17 Abs. 1 DSG NRW, die erforderlichen zusätzlichen Maßnahmen richten sich nach § 17 Abs. 2 S. 1, § 15 DSG NRW [20].

Für die Datenverarbeitung in Krankenhäusern sehen § 6 Abs. 2 S. 1, 2 GDSG NW Rechtsgrundlagen für die „Nutzung“ von Patientendaten zu wissenschaftlichen Forschungszwecken vor, einschließlich weiterer Anforderungen an Anonymisierung, Pseudonymisierung und Veröffentlichung nach § 6 Abs. 3–5 GDSG NW. Die „Nutzung“ betrifft jedoch die Verarbeitungsphase nach der Erhebung, nicht die initiale Datenerhebung.^7^ Außer der „Nutzung“ ist die Verarbeitung von Patientendaten zu wissenschaftlichen Forschungszwecken, einschließlich der Erhebung nach §§ 10 Abs. 2, 4, 6 Abs. 1 GDSG NW, lediglich mit einer Einwilligung der betroffenen Personen gestattet. Anders als andere LKHG^8^ enthält das GDSG NW damit keine Vorschrift, die die Erhebung von Patientendaten zu wissenschaftlichen Forschungszwecken ohne Einwilligung gestatten würde [21]. Eine solche Einwilligung ist insofern in jedem Fall erforderlich.

## Weiterverarbeitung von MRT-Gehirnscans

Von der initialen Verarbeitung zum Erhebungszweck zu unterscheiden ist die Weiterverarbeitung, also die anschließende Verarbeitung personenbezogener Daten zu einem anderen Zwecke als dem Erhebungszweck, Art. 6 Abs. 4 DSGVO. Der Zweckbindungsgrundsatz des Art. 5 Abs. 1 lit. b DSGVO fordert, dass die Zwecke der Verarbeitung im Voraus konkret bestimmt werden, allgemeine Bezeichnungen wie „wissenschaftliche Forschung“ oder „Patientenbehandlung“ genügen nicht [20].

In der Konsequenz stellt die Verarbeitung im Zusammenhang mit der Patientenbehandlung oder einem anderen Forschungsvorhaben gewonnener Daten zu (anderen) Forschungsvorhaben eine Zweckänderung und Weiterverarbeitung dar [22, 23]. Art. 5 Abs. 1 lit. b DSGVO gestattet die zweckändernde Weiterverarbeitung zu mit dem Primärzweck kompatiblen Sekundärzwecken. Art. 6 Abs. 4 DSGVO nennt konkretisierte Kriterien für diese Kompatibilitätsprüfung. Privilegiert werden durch Art. 5 Abs. 1 lit. b DSGVO Zweckänderungen zu Zwecken der wissenschaftlichen Forschung nach Art. 89 Abs. 1 DSGVO, bei der eine Zweckvereinbarkeit vermutet wird. Davon abgesehen ist eine zweckändernde Weiterverarbeitung auch mit (erneuter) Einwilligung zulässig. Beide Varianten des Art. 6 Abs. 4 DSGVO werden im Folgenden dargestellt.

### Zweckändernde Weiterverarbeitung mit (erneuter) Einwilligung.

Zulässig ist die Weiterverarbeitung der MRT-Gehirnscans im Kontext konkreter wissenschaftlicher Forschungsvorhaben jedenfalls dann, wenn die Weiterverarbeitung auf einer entsprechenden Einwilligung der betroffenen Person im Sinne des Art. 9 Abs. 2 lit. a DSGVO in Verbindung mit §§ 6 Abs. 1, 4 GDSG NW erfolgt. Solche Einwilligungen können Forschende z. B. im Krankenhaus oder bei anderen Leistungserbringern bereits bei der Datenerhebung einholen; Art. 9 Abs. 2 lit. a, Art. 6 Abs. 1 lit. a DSGVO gestatten ausdrücklich die Einwilligung in mehrere Verarbeitungszwecke, solange diese hinreichend bestimmt ist.

Unter der Nutzung des MII-Einwilligungsformulars ist auch die Einholung von Broad-Consent-Einwilligungen in weniger konkret bestimmte Verarbeitungszwecke im Kontext von Forschungsvorhaben möglich (s. oben). Eingeholt werden können auch spezifische Einwilligungen für die Verarbeitung im Rahmen konkreter Forschungsvorhaben, die sich erst zu einem späteren Zeitpunkt ergeben, allerdings im Zuge einer (Re)Kontaktierung der betroffenen Personen, wobei es auch hierzu einer Rechtsgrundlage, etwa einer vorherigen Einwilligung der betroffenen Person, bedarf.

### Zweckändernde Weiterverarbeitung ohne Einwilligung auf gesetzlicher Grundlage.

Die Anforderungen an die Zulässigkeit der Weiterverarbeitung auf gesetzlicher Grundlage ohne Einwilligung nach Art. 6 Abs. 4 DSGVO sind umstritten. Wegen des Wortlautes des ErwGr. 50 S. 2 DSGVO wird teilweise davon ausgegangen, die Weiterverarbeitung könne auf die Erlaubnisgrundlage der Ersterhebung gestützt werden, sofern eine Kompatibilitätsprüfung anhand der in Art. 6 Abs. 4 DSGVO genannten Kriterien positiv ausfällt [24, 25]. Nach (wohl) überwiegender Auffassung erfordert die Weiterverarbeitung jedoch in jedem Fall eine gesonderte Erlaubnisgrundlage zusätzlich zu einer positiven Kompatibilitätsprüfung [26–28]. Unter Berücksichtigung des ErwGr. 51 S. 5 DSGVO kommt Art. 6 Abs. 4 DSGVO richtigerweise auch bei der Verarbeitung sensibler Kategorien personenbezogener Daten nach Art. 9 Abs. 1 DSGVO zur Anwendung ([29], anders: [30]).

### Erlaubnistatbestände für zweckändernde Weiterverarbeitung

Art. 6 Abs. 4 DSGVO normiert keine allgemeine Öffnungsklausel für mitgliedsstaatliches Recht, durch welches die zweckändernde Weiterverarbeitung gestattet wird, sondern ist im Zusammenhang mit Art. 6 Abs. 2, 3 DSGVO zu lesen, der den Mitgliedsstaaten die Schaffung zusätzlicher Regelungen in Bezug auf die Erlaubnisgrundlagen der Art. 6 Abs. 1 lit. c und e DSGVO gestattet [27].

Für Normen, die (auch) die Weiterverarbeitung sensibler personenbezogener Daten gestatten, können die Mitgliedsstaaten aber auf die Öffnungsklauseln des Art. 9 Abs. 2 DSGVO zurückgreifen. Solche Erlaubnistatbestände für die zweckändernde Weiterverarbeitung von MRT-Bildern als Gesundheitsdaten umfassen die Art. 9 Abs. 2 lit. j DSGVO (Art. 6 Abs. 1 lit. f DSGVO) in Verbindung mit § 6 Abs. 2 S. 1, 2 GDSG NW als bereichsspezifische Norm für die Verarbeitung von Patientendaten in Krankenhäusern sowie subsidiär §§ 17 Abs. 1, 15 DSG NRW bzw. §§ 27 Abs. 1 S. 1, 22 BDSG.

#### Datenverarbeitung zu Zwecken der wissenschaftlichen Forschung, § 6 Abs. 2 S. 1 GDSG NW

§ 6 Abs. 2 S. 1 GDSG NW gestattet dem wissenschaftlichen Personal in Krankenhäusern die Nutzung von Patientendaten, auf die sie im Rahmen ihrer Tätigkeiten ohnehin Zugriff haben, ohne Einwilligung zu Zwecken der wissenschaftlichen Forschung. Weitere qualifizierende Anforderungen benennt die Norm nicht; § 6 Abs. 4 GDSG NW bestimmt aber jedenfalls, dass Daten zu pseudonymisieren sowie zu anonymisieren sind, sobald der Forschungszweck dies gestattet.

Angesichts der hohen Anforderungen, die Art. 9 Abs. 2 lit. j, Art. 89 Abs. 1 DSGVO an nationale Umsetzungsgesetze stellen, kann hinterfragt werden, ob § 6 Abs. 2 S. 1 GDSG NW unionsrechtskonform ist. Jedenfalls findet die Norm nur insoweit Anwendung, wie Forschung innerhalb eines Krankenhauses betrieben wird, bei welcher ohnehin „vorhandene“ Patientendaten zweckändernd zu Zwecken der wissenschaftlichen Forschung verwendet werden. Die Übermittlung von Patientendaten an andere Einrichtungen oder Stellen inner- oder außerhalb des Krankenhauses kann aber nicht auf § 6 Abs. 2 S. 1 GDSG NW, wohl aber auf § 6 Abs. 2 S. 2 GDSG NW gestützt werden.^9^

#### Forschung Dritter, § 6 Abs. 2 S. 2 GDSG NW

§ 6 Abs. 2 S. 2 GDSG NW gestattet die Verarbeitung von Patientendaten zu Forschungszwecken ohne Einwilligung, einschließlich der Übermittlung an Stellen außerhalb von Krankenhäusern [31]. Hierfür bedarf es neben der Erforderlichkeit der Verarbeitung für den Zweck des Forschungsvorhabens (§ 6 Abs. 2 S. 2 Nr. 1) und der Unmöglichkeit der Einholung der Einwilligung von der betroffenen Person (§ 6 Abs. 2 S. 2 Nr. 3) eines erheblichen Überwiegens der Interessen an der Verarbeitung gegenüber den Geheimhaltungsinteressen der betroffenen Personen (§ 6 Abs. 2 S. 2 Nr. 2). Ähnliche Anforderungen stellen auch § 17 Abs. 1 DSG NRW sowie § 27 Abs. 1 S. 1 BDSG: § 27 Abs. 1 S. 1 BDSG macht wie § 6 Abs. 2 S. 2 GDSG NW die Zulässigkeit der Verarbeitung davon abhängig, dass die Verarbeitung erforderlich ist und die Interessen des Verantwortlichen an der Verarbeitung die Interessen betroffener Personen an deren Ausschluss „erheblich“ überwiegen.

Nach § 17 Abs. 1 DSG NRW soll die erforderliche Verarbeitung hingegen dann zulässig sein, wenn die Interessen betroffener Personen gegenüber den Interessen an der Verarbeitung nicht überwiegen. Im Vergleich der Normen sind hier also geringere Anforderungen an die Datenverarbeitung gestellt.

Eine § 6 Abs. 2 S. 2 Nr. 3 GDSG NW entsprechende Vorgabe, dass die Einholung der Einwilligung von der betroffenen Person unmöglich ist, sehen weder § 17 Abs. 1 DSG NRW noch § 27 Abs. 1 S. 1 BDSG vor. § 17 Abs. 1 DSG NRW, § 27 Abs. 1 S. 1 BDSG werden bei der Datenverarbeitung im Krankenhauskontext regelmäßig durch speziellere Regelungen der Landeskrankenhausgesetze wie § 6 Abs. 2 GDSG NW verdrängt und kommen entsprechend nicht zur Anwendung.

Die nach § 6 Abs. 2 S. 2 Nr. 2 GDSG NW, aber auch nach § 17 Abs. 1 DSG NRW und § 27 Abs. 1 S. 1 BDSG erforderliche umfassende Abwägung hat zu erfolgen zwischen den Interessen der betroffenen Personen am Unterbleiben der Verarbeitung und den Interessen der Allgemeinheit an der Durchführung der Verarbeitung im Einzelfall, wobei auch zu berücksichtigen ist, welche Maßnahmen zur Senkung von Verarbeitungsrisiken ergriffen wurden (s. unten).

##### Interessen am Unterbleiben der Verarbeitung.

Interessen betroffener Personen, die gegen eine Datenverarbeitung sprechen, sind etwa deren Interesse am Daten- und/oder Privatsphäreschutz, vgl. Art. 8, 7 Grundrechte-Charta der EU (GRCh) und daran, physische, immaterielle oder materielle Nachteile aus der Verarbeitung zu vermeiden oder zumindest gering zu halten.

Mit der Verarbeitung von Gesundheitsdaten gehen wegen ihrer Sensibilität besondere Risiken einher, vgl. ErwGr. 51 S. 1 DSGVO. Speziell bei MRT-Gehirnscans besteht das hohe Risiko der Identifizierung der betroffenen Personen (s. oben). Ist die Verarbeitung durch Dritte mit der Übermittlung der Daten verbunden, nehmen wegen der steigenden Schwierigkeiten, die absprachegemäße Verarbeitung zu überwachen und zu kontrollieren, auch Risiken der ungewollten oder unbefugten Nutzung und Offenlegung zu. Betroffene Personen werden aufgrund solcher und weiterer mit der Verarbeitung verbundener Risiken häufig Interesse am Ausschluss der Verarbeitung haben, insb. wenn eine Übermittlung der Daten an Dritte stattfinden soll.

##### Interessen an der Durchführung der Verarbeitung.

Für die Verarbeitung im Interesse der Allgemeinheit sprechen etwa die Erkenntnisse, die aus der Datenverarbeitung zu Forschungszwecken für die individuelle sowie gesamtgesellschaftliche Gesundheitsversorgung gewonnen werden können [32]. Dies ist ein unionsrechtlich gefördertes Ziel, Art. 179 AEUV; die Forschungsfreiheit ist überdies grundrechtlich geschützt, Art. 13 GRCh, Art. 5 Abs. 3 Grundgesetz (GG).

Vielfach überschneiden sich die individuellen Interessen der Forschenden an der Durchführung des Forschungsvorhabens mit den Interessen der Allgemeinheit. Das von § 6 Abs. 2 S. 2 Nr. 2 GDSG NW sowie § 27 Abs. 1 S. 1 BDSG geforderte „erhebliche“ Überwiegen dieser Interessen ist eine hohe Hürde, kann aber etwa vorliegen, wenn die Durchführung des Forschungsvorhabens erhebliche Verbesserungen für die Gesundheit der Bevölkerung mit sich bringt.

##### Zusätzliche Maßnahmen zur Senkung der Risiken der Verarbeitung.

In der Interessenabwägung zu berücksichtigen sind auch technische oder organisatorische Maßnahmen, die zur Senkung von Verarbeitungsrisiken beitragen. § 6 Abs. 3–6 GDSG NW sehen solche vor, namentlich die Protokollierung der Übermittlungstätigkeiten (Abs. 3), die Pseudonymisierung und Anonymisierung der Patientendaten, einschließlich der getrennten Speicherung der Reidentifizierungsmerkmale bis zu deren Löschung, sobald der Forschungszweck dies zulässt (Abs. 4), die Beschränkung der Veröffentlichung von Forschungsergebnissen auf anonymisierte Daten, soweit keine Einwilligung der betroffenen Person vorliegt (Abs. 5), sowie die vertragliche Verpflichtung des Empfängers übermittelter Daten zur Einhaltung des Verarbeitungszwecks, der Vorgaben der Abs. 4, 5, Gewähr der Einsicht für die für die übermittelnde Stelle zuständigen Aufsichtsbehörden sowie Einrichtung technisch-organisatorischer Maßnahmen zum Nachweis der Erfüllung der Verpflichtung (Abs. 6).

### Kompatibilitätsprüfung, Art. 6 Abs. 4 DSGVO

Erforderlich für die Weiterverarbeitung nach Art. 6 Abs. 4 DSGVO ist weiterhin die Vereinbarkeit des Sekundärzwecks der Verarbeitung mit dem Primärzweck der Erhebung. In der Regel wird die Kompatibilität bei einer Weiterverarbeitung zu wissenschaftlichen Forschungszwecken nach Art. 5 Abs. 1 lit. b, Art. 89 Abs. 1 DSGVO vermutet. Ist dies nicht der Fall, ist der nicht abschließende („unter anderem“) Kriterienkatalog des Art. 6 Abs. 4 DSGVO zu beachten, z. B. ob es sich um besondere Kategorien personenbezogener Daten handelt oder ob geeignete Maßnahmen zur Sicherheit der Verarbeitung ergriffen wurden. Fällt die Kompatibilitätsprüfung positiv aus, können die personenbezogenen Daten zu dem benannten Sekundärzweck weiterverarbeitet werden.

## Ausblick: Einsatz von Datentreuhändern zur Reduzierung der Verarbeitungsrisiken

Der Einsatz sicherer Verarbeitungsumgebungen, wie sie Datentreuhänder bereithalten, trägt zur Reduzierung von Verarbeitungsrisiken bei und ist daher bei oben genannten Abwägungsentscheidungen zugunsten der Datenverarbeitung zu berücksichtigen.

Indem die Verarbeitungsrisiken erheblich reduziert werden, kann die Interessenabwägung nach § 6 Abs. 2 S. 2 GDSG NW bzw. § 17 Abs. 1 DSG NRW oder § 27 Abs. 1 S. 1 BDSG trotz der hohen Anforderungen eher zugunsten der nunmehr weniger risikobehafteten Verarbeitung ausfallen. Auch die in § 15 DSG NRW bzw. § 22 Abs. 2 S. 2 BDSG genannten technischen und organisatorischen Maßnahmen können durch solche Datentreuhänder umgesetzt werden. Diese können damit wesentlich dazu beitragen, Daten auf gesetzlicher Grundlage verarbeiten zu dürfen.

Datentreuhänder als unabhängige Stelle zur Durchführung sicherer Pseudonymisierung werden etwa im Kontext von Biodatenbanken diskutiert^10^ [34, 35]. Bei solchen können entsprechend Art. 4 Nr. 5 DSGVO die Identifizierungsmerkmale getrennt gespeichert und so durch technisch-organisatorische Maßnahmen die ungewollte oder unbefugte Reidentifizierung der Betroffenen verhindert werden. Ebenso können Datentreuhänder als Anonymisierungsinstanz tätig werden, was vor dem Hintergrund der hohen Anforderungen an die Verarbeitung personenbezogener Daten und der teils erheblichen Rechtunsicherheiten in Bezug auf die Anonymisierung für Verantwortliche entlastend wirken könnte.^11^

Gerade für MRT-Bilddateien ist dies von Relevanz, weil gängige Methoden, wie die Entfernung unmittelbar identifizierender Faktoren wie Namen, Alter, Geschlecht etc.,^12^ nicht zu einer Anonymisierung der Gehirnscans führen (s. oben). Zwar kann die Anonymisierung durch das „Herausschneiden“ der Gesichtspartie erreicht werden (sog. Defacing; [40]), dies bindet jedoch nicht unerhebliche technische und personelle Ressourcen. Insofern ist der Einsatz von Datentreuhändern zur Pseudonymisierung oder Anonymisierung durchaus eine lohnende Überlegung.

Anonymisierte oder pseudonymisierte Daten können zur anschließenden Verarbeitung an einen weiteren unabhängigen Datentreuhänder gegeben werden, der eine sichere Umgebung bereitstellt, in der die Verarbeitung erfolgt (In-situ-Verarbeitung). Hierbei hätten die Datengeber selbst keinen Zugriff auf die Gesundheitsdaten, sondern die Verarbeitung könnte entsprechend ihren Anweisungen ausschließlich in der Verarbeitungsumgebung durch den Datentreuhänder erfolgen. Lediglich die Verarbeitungsergebnisse, nicht jedoch die Rohdaten, würden im Anschluss an die Bereitstellung und nach Anweisung der Datengeber-Dritten, etwa anderen Kliniken, zur weiteren forschungsbezogenen Nutzung ausgespielt werden.

Denkbar ist auch eine Kombination von Datensätzen aus unterschiedlichen Quellen, bei der jeweils sichergestellt wird, dass die Forschungspartnerinnen und -partner keinen Zugriff auf die Rohdaten der jeweils anderen Quelle erlangen. Hierzu könnten auch eigene Algorithmen der Kliniken eingesetzt werden, sodass die Verarbeitung entsprechend ihren Bedürfnissen erfolgen kann.

Nach einer für die Verarbeitung erforderlichen kurzfristigen Speicherung bei dem Datentreuhänder könnten die Daten anschließend gelöscht werden. Der Datentreuhänder könnte hierbei als weisungsabhängiger Auftragsverarbeiter der Datengeber bzw. -nutzer eingebunden werden, sodass es für die Rechtmäßigkeit der Verarbeitung durch diesen auf die Rechtmäßigkeit der Verarbeitung für die Datengeber und -nutzer als Verantwortliche ankäme.

Weil der Einsatz eines Datentreuhänders gerade zur Reduzierung der Verarbeitungsrisiken beiträgt, können Verarbeitungstätigkeiten wie die Anonymisierung, Pseudonymisierung, kurzfristige Zwischenspeicherung, aber auch die Auswertung der Daten eher nach Art. 9 Abs. 2 lit. j DSGVO in Verbindung mit nationalem Recht gerechtfertigt werden als eine Datenverarbeitung ohne solche Datentreuhänder. Die Datenempfänger haben keinen Zugriff auf die (Roh‑)Daten, sondern ausschließlich auf die Verarbeitungsergebnisse, daher sind die Möglichkeiten der unberechtigten Weiterverarbeitung der Daten zu eigenen Zwecken äußerst beschränkt.

Werden Datentreuhänder als weisungsuntergebene Auftragsverarbeiter der Datengeber eingesetzt, werden deren Möglichkeiten der Kontrollausübung über die Datenverwendung auch bei einer Bereitstellung an andere erhöht. Ermöglicht wird eine Kombination von Datensätzen, ohne dass tatsächlich ein technisch-faktischer Zugang zu diesen gewährt werden muss. Auch Risiken des unbefugten Zugriffs durch Dritte werden reduziert.

## Fazit

Der Einsatz von Datentreuhänder, die Pseudonymisierungs- und Anonymisierungsdienstleistungen erbringen sowie sichere Verarbeitungsumgebungen bereitstellen, kann die mit der Verarbeitung von Gesundheitsdaten verbundenen Risiken erheblich reduzieren und hierdurch zur Wahrung der Interessen betroffener Personen beitragen. Die nach § 6 Abs. 2 S. 2 GDSG NW, § 17 Abs. 1 DSG NRW oder § 27 Abs. 1 BDSG erforderliche Interessenabwägung fällt insoweit eher zugunsten der Datenverarbeitung aus. Es bleibt aber dabei, dass diese Interessenabwägung erfolgen muss und nie sicher beurteilt werden kann, dass nicht doch bei einigen der in der Treuhand verarbeiteten Daten die Interessenabwägung zugunsten der Betroffenen ausfällt.

Auch durch den Einsatz einer Datentreuhand kann insofern die Rechtsunsicherheit, die auch in Fällen der Forschung im Gemeinwohlinteresse besteht und diese hemmt, nicht gänzlich ausgeräumt werden. Erforderlich ist, dass der Gesetzgeber die Chancen und Möglichkeiten der Datentreuhand erkennt und die Datenverarbeitung in derartigen Treuhandumgebungen sowie die Datenübermittlung in die Datentreuhand auf eine Rechtssicherheit gewährleistende Forschungsklausel stellt.

Auch ohne eine solche Forschungsklausel wird die Rechtssicherheit bei der Sekundärnutzung von Daten, wie z. B. MRT-Gehirnscans, zu Forschungszwecken aber zumindest erhöht. Forschungsvorhaben, die auf einen Austausch von Datenbeständen mit anderen Einrichtungen angewiesen sind, könnten hier profitieren.

Perspektivisch könnten solche Datentreuhänder auch eine aktivere Rolle einnehmen, etwa als eine marktplatzähnliche Plattform, die geneigte Kliniken mit Patientendatenbeständen und andere Einrichtungen zur gemeinsamen Forschung zusammenbringt. Sie könnten aktiv die Einhaltung der von den Datengebern gestellten Datenverarbeitungskonditionen durchsetzen, bei Vertragsverhandlungen mitwirken sowie die Prüfung des Vorliegens etwaiger Rechtsgrundlagen für die Datenverarbeitung wahrnehmen. Mit solchen Funktionen wären die Datentreuhänder aber nicht nur selbst datenschutzrechtliche Verantwortliche im Sinne des Art. 4 Nr. 7 DSGVO, sondern wohl auch Datenvermittlungsdienste im Sinne des Art. 2 Nr. 11, Art. 10 lit. a Data Governance Act (DGA), woraus sich weitere rechtliche An- und Herausforderungen ergeben.

Aber auch in ihrer „Basisfunktion“ der sicheren Verarbeitungsräume stellen Datentreuhänder eine sinnvolle Ergänzung zu Forschungsdatenzentren und der Zugangsstellen für Gesundheitsdaten nach Art. 37 des European-Health-Data-Space-Verordnungsentwurfs (EHDS-VO-E)^13^ dar, denn schließlich ermöglichen sie eine Zusammenführung und Auswertung individuell bestimmbarer Datenbestände und gewähren – anders als die Forschungsdatenzentren – potenziell auch Zugang zu privat gehaltenen Daten.

## Footnotes

1 Umfassenden Überblick zu den Erlaubnisgrundlagen für die Verarbeitung von Gesundheitsdaten bietet etwa: [1, Rn. 38 ff.]. Eine Auflistung von Befugnisnormen speziell für Krankenhäuser findet sich bei: [2, S. 45 ff.].

2 Für diesen Aufsatz beispielhaft herausgegriffen werden soll das Gesundheitsdatenschutzgesetz Nordrhein-Westfalen (GDSG NW), welches Vorgaben zur Verarbeitung von personenbezogenen Daten, die Patientendaten sind, in Krankenhäusern im Land Nordrhein-Westfalen regelt. Das GDSG NW wurde, wie auch andere LKHG, bisher nicht an die Rechtslage unter der DSGVO angepasst, wodurch teilweise Rechtsfragen hinsichtlich der Unionsrechtskonformität entstehen, die hier aber nicht im Einzelnen erörtert werden können. Im Überblick über den „Entwicklungsstand“ der Reformierung der LKHG bzw. LGDSG: [2, S. 41 ff.].

3 Das Einwilligungsformular ist abrufbar unter: https://www.medizininformatik-initiative.de/sites/default/files/2020-04/MII_AG-Consent_Einheitlicher-Mustertext_v1.6d.pdf [4]. Zur Kritik wegen der hohen Komplexitätsanforderungen s. [5].

4 Vgl. zur Begriffsdefinition sicherer Verarbeitungsumgebungen in Art. 2 Nr. 20 Data Governance Act (DGA): [6, ab Rn. 161].

5 Beachtenswert ist die Darstellung des Verhältnisses zwischen Art. 9 und Art. 6 DSGVO in ErwGr. 37 European-Health-Data-Space-Verordnungsentwurf (EHDS-VO-E), nach dem die Entwurfsverfassenden davon auszugehen scheinen, lediglich Art. 6 DSGVO normiere Erlaubnistatbestände und Art. 9 DSGVO zusätzliche Anforderungen. Damit scheinen sich die Entwurfsverfassenden der letztgenannten Auffassung anzuschließen.

6 Nach dem Wortlaut gestattet Art. 9 Abs. 2 lit. a DSGVO mitgliedsstaatliche Regelungen, nach denen das Verbot des Art. 9 Abs. 1 DSGVO durch die Einwilligung nicht aufgehoben werden kann. Neben solchen Einwilligungsausschlüssen gestattet Art. 9 Abs. 2 lit. a DSGVO nach überwiegender Auffassung aber auch die Bestimmung zusätzlicher Anforderungen an die Einwilligung als „Minus-Maßnahmen“ durch mitgliedsstaatliches Recht (hierzu [17, S. 49 f.]).

7 Dies legen der Vergleich der Formulierungen in § 6 Abs. 1 und in Abs. 2 GDSG NW sowie die Betrachtung der historischen Definition der Begrifflichkeiten „Übermittlung“ und „Verarbeitung“ (§ 6 Abs. 1 GDSG NW) und „Nutzung“ (§ 6 Abs. 2 S. 1 GDSG NW) nahe. Das GDSG NW enthält keine eigenen Begriffsdefinitionen, historisch konnte nach der Subsidiaritätsklausel des § 3 S. 1 GDSG NW auf die Begriffsdefinitionen des § 3 DSG NRW 1988 zurückgegriffen werden, das inzwischen zugunsten des nunmehr geltenden DSG NRW aufgehoben wurde. § 3 Abs. 2 S. 1 DSG NRW 1988 definierte die Datenverarbeitung als „das Erheben, Speichern, Verändern, Übermitteln, Sperren, Löschen und Nutzen personenbezogener Daten“. Die einzelnen Verarbeitungsformen wurden in § 3 Abs. 2 S. 2 DSG NRW 1988 jeweils eigenständig legaldefiniert und waren entsprechend voneinander abzugrenzen. Die Nutzung wurde nach § 3 Abs. 2 S. 2 Nr. 7 DSG NRW 1988 als „jede sonstige Verwendung personenbezogener Daten“ definiert und hat insofern einen Auffangtatbestand für alle Verarbeitungsformen geschaffen, die keine Erhebung, Speicherung, Veränderung, Übermittlung, Sperrung oder Löschung waren. Diesen historischen Kontext und die Wortwahl in § 6 Abs. 2 S. 1 GDSG NW berücksichtigend ist es naheliegend, dass der GDSG-Gesetzgeber lediglich für die spezifische Verarbeitungsform der „Nutzung“ eine Ausnahme vom allgemeinen Einwilligungsvorbehalt des § 6 Abs. 1 GDSG NW treffen wollte.

8 So etwa § 25 Abs. 1 LKHG Berlin, § 37 Abs. 1 S. 2 LKHG Rheinland-Pfalz oder § 27 Abs. 4 S. 1 LKHG Thüringen, die jeweils weitreichender von der „Verarbeitung“ von Patientendaten sprechen.

9 Siehe Fußnote 7 zur Abgrenzung der unterschiedlichen Verarbeitungsschritte unter Berücksichtigung der historischen Auslegung des GDSG NW anhand der historischen Begriffsdefinitionen des § 3 Abs. 2 S. 2 DSG NRW 1988. § 5 Abs. 1 S. 2 GDSG NW enthält eine eigene, § 3 Abs. 2 S. 2 Nr. 4 DSG NRW 1988 ergänzende Definition der Übermittlung, nach der „auch die Weitergabe von Patientendaten an Personen in anderen Organisationseinheiten innerhalb der Einrichtung oder öffentlichen Stelle, sofern diese Organisationseinheiten nicht unmittelbar mit Untersuchungen, Behandlungen oder sonstigen Maßnahmen nach § 2 Abs. 1 befaßt sind“, als Übermittlung gilt. Zum Verständnis des § 6 Abs. 2 S. 1 GDSG NW als Norm für ausschließlich interne Forschung ohne Übermittlung s. a.: [31, S. 31].

10 Im Überblick zu unterschiedlichen Datentreuhandmodellen in der medizinischen Forschung: [33, S. 807].

11 Zu den Anforderungen an eine Anonymisierung unter dem zunehmenden Einsatz künstlicher Intelligenz: [36]. Zur unterschiedlichen rechtlichen Beurteilung der Anonymisierung als Löschung im Sinne des Art. 17 DSGVO: [37], zur unterschiedlichen Beurteilung der Anonymisierung als Datenverarbeitung nach Art. 4 Nr. 2 DSGVO: [38].

12 Im Überblick zu solchen siehe etwa: [39, S. 13 ff.].

13 Vorschlag für eine Verordnung des Europäischen Parlaments und des Rates über den europäischen Raum für Gesundheitsdaten, COM (2022) 197 final.
